# Is There a Difference in Overweight and Obesity Between Christian Orthodox Fasters and Non-Fasters? A Cross-Sectional Study in Northern Greece

**DOI:** 10.3390/nu17203308

**Published:** 2025-10-21

**Authors:** Nikolaos E. Rodopaios, Aikaterini Apostolopoulou, Alexandra-Aikaterini Koulouri, Sousana K. Papadopoulou, Petros Skepastianos, Maria Hassapidou, Zoi Tsimtsiou, Antony G. Kafatos

**Affiliations:** 1 Department of Social Medicine, Preventive Medicine and Nutrition, Medical School, University of Crete, 71110 Heraklion, Greece; nikow1966@yahoo.gr (N.E.R.); alexkoulou@yahoo.com (A.-A.K.); kafatos@med.uoc.gr (A.G.K.); 2 Department of Hygiene, Social-Preventive Medicine and Medical Statistics, School of Medicine, Aristotle University of Thessaloniki, 54124 Thessaloniki, Greece; aapostoo@auth.gr; 3 Department of Nutritional Sciences and Dietetics, School of Health Sciences, International Hellenic University, 57400 Thessaloniki, Greece; sousana@ihu.gr (S.K.P.); mnhas@ihu.gr (M.H.); 4 Department of Biomedical Sciences, School of Health Sciences, International Hellenic University, 57400 Thessaloniki, Greece; pskep@otenet.gr

**Keywords:** Christian Orthodox fasting, obesity, overweight, nutrients, religious fasting

## Abstract

Objectives: The aim of this study was to assess nutrient intake among individuals adhering to the Christian Orthodox Church (COC) fasting and to investigate potential differences in dietary intake according to Body Mass Index (BMI) classification. Methods: This cross-sectional study enrolled participants through announcements at public universities, churches, and monasteries, targeting both urban and religious adult populations. A total of 228 adults with a BMI exceeding 25 kg/m^2^ were enrolled. Of these, 121 had followed COC fasting practices for at least 10 years or since childhood, while 107 non-fasters were age-matched. Exclusion criteria included age under 18 years, refusal to provide consent, absence from measurements, non-communicable diseases, food allergies, pregnancy, or lactation. Results: Overweight and obesity rates were similar in both groups. Furthermore, there were no statistically significant differences in body composition measurements [body fat %, fat mass (kg), fat free mass (kg), waist circumference]. Diastolic and systolic blood pressure was significantly higher in non-fasters. Non-fasters reported higher intake of sugar, dietary protein, fats (saturated and polyunsaturated), and cholesterol. Fasters consumed lower amounts of vitamin A, vitamins B (B2, B3, B6, B12, folate, pantothenic acid), iron, phosphorus, sodium, zinc, and calcium. Serum folic acid levels were higher, and fasting glucose and phosphorus levels were lower in fasters. Distinct dietary patterns were observed between groups, with fasters consuming more fish and traditional plant-based foods, while non-fasters consumed higher amounts of meat, dairy products, and alcohol. Conclusions: COC fasting is associated with favorable dietary and metabolic profiles, including improved glucose regulation. However, its impact on weight status appears limited.

## 1. Introduction

The Christian Orthodox Church (COC), which is followed by Orthodox Christians [1], prescribes a fasting practice that involves avoiding meat, dairy products, and eggs for approximately 180–200 days each year, according to its distributed fasting periods. The total days of COC fasting range from 159 to 197 (average, 178) and include five main periods, and two important religious days, Wednesdays, and Fridays [2]. During these times, the consumption of fish is allowed on some fasting days, while seafoods and snails are allowed on all fasting days, while the intake of fruits, vegetables, legumes, and cereals is encouraged. This fasting tradition, transitioning from a plant-based to a vegetarian diet, is regarded as a cornerstone of the Mediterranean diet [3]. COC fasting is often equated with the Mediterranean Diet (MD) and regarded as a vital component of this eating pattern. It is also recognized as a form of periodic vegetarianism.

The similarities between the fasting practices of the COC with healthy dietary patterns, such as MD, logically lead to an association between fasting and a positive impact on human health. Prior studies have found that individuals who adhere to Orthodox fasting tend to have a more favorable lipid profile and a lower body mass index (BMI) compared to those who do not practice fasting [4,5]. A recent review also agrees with these findings, as it suggests that following COC fasting guidelines may improve lipid profile and body composition, without causing iron deficiency or reductions in bone mineral density; however, available evidence remains limited [3]. Weight fluctuations, particularly weight loss, frequently occur during extended fasting periods and are often associated with fat loss [6,7,8,9]. It remains debatable whether weight loss is sustained. The positive effects of COC fasting on the adipokine profile [10,11] and glucose homeostasis in both monastic and general populations [12] has been stated, highlighting their potential as markers for preventing cardiovascular dyshomeostasis.

Regarding micronutrient intake profile of COC fasting, severe hypovitaminosis D has been shown to be prevalent among COC fasters [13]. Also, the abstinence from animal product consumption has been shown to lead to inadequate calcium and magnesium intake [4,5,6]. However, this religious fasting pattern is characterized as a low-sodium diet, offering a potentially beneficial micronutrient profile for cardiovascular disease prevention, particularly in terms of sodium and potassium intake [14]. Additionally, a notable advantage of COC fasting is high dietary fiber intake [13]. On the other hand, intake levels of iron are expected to be inadequate in fasting populations [4,15].

COC fasting has been shown to influence hematological parameters. Increases in INR and decreases in aPTT, blood tests used to measure how long it takes for blood to clot to monitor patients taking vitamin K antagonist anticoagulants or heparin, respectively, suggest effects on both coagulation pathways, possibly linked to the fasting diet rich in nuts, vegetables, and seeds [16]. Reduction in total white blood cells and granulocytes, alongside higher lymphocyte counts after the 48-day pre-Easter fast has also been reported, along with a hematocrit, hemoglobin, and mean corpuscular volume (MCV) decrease, remaining though within normal ranges [16]. Overall, fasting appears to enhance antioxidant defenses and may contribute to cardiovascular disease prevention [16,17]. Other studies reported higher intakes of dietary fiber and antioxidant vitamins, and lower intakes of saturated fat and dietary cholesterol during fasting periods, compared with non-fasting adults and adolescents from the same region [5,18]. Such dietary patterns are associated with improved lipid profiles, including lower total and LDL cholesterol, as well as better glycemic control through enhanced insulin sensitivity. These effects may contribute to a reduced risk of type 2 diabetes and cardiovascular disease, thereby highlighting the potential long-term cardiometabolic benefits of COC fasting [19,20,21]. Regarding potential negative effects of COC fasting, although there are studies indicating inadequacies of certain micronutrients during fasting periods [19,20], a systematic review by Koufakis et al. [22] did not reach firm conclusions. Evidence on reduced intakes of vitamin B12, vitamin D, and minerals such as calcium remains contradictory.

Given the previously reported health benefits of COC fasting, it is crucial to further explore its effects in populations at increased risk for chronic disease. Individuals with elevated Body Mass Index (BMI) represent a key target group, as overweight and obesity are strongly associated with metabolic disturbances, inflammation, and cardiovascular risk [23,24]. For these populations, even modest dietary modifications may have disproportionate health benefits. Therefore, investigating the possible effects of practices associated with COC fasting in individuals with increased BMI could provide valuable insights into dietary recommendations for weight management and disease prevention in high-risk populations [25,26].

The aim of this study was to assess the nutrient intake levels of COC fasters and non-fasters with overweight and obesity and to examine potential differences based on their BMI.

## 2. Materials and Methods

### 2.1. Study Design and Study Population

This is a cross-sectional study that enrolled volunteers through a call in public universities, monasteries, and churches in Thessaloniki from 2013 until 2015. The study protocol was approved by the Bioethics Committee of the Alexander Technological Educational Institute of Thessaloniki (ΔΦ 31-5/5679, date 17 December 2013). Informed consent was obtained from all the participants, and withdrawal was feasible at any time.

A total of 228 participants with overweight and obesity were enrolled according to our inclusion criteria, 121 followed the COC fasting recommendations, including 180–200 days of the year since their childhood or at least the previous 10 years. The chronic duration of slightly over a decade was deemed sufficient to draw reliable conclusions regarding the effects of fasting on human health. To ensure that the data for fasters were representative of typical fasting behavior throughout the year, measurements were not conducted during extended fasting periods (e.g., religious holidays) or during times of unrestricted eating. Instead, all anthropometric measurements and blood sampling were performed exclusively during routine fasting days, namely Wednesdays and Fridays. The remaining 107 participants did not follow COC fasting and were age-matched with the fasting group to ensure reliable comparisons. The exclusion criteria included: (a) participants younger than 18 years, (b) failure to provide written informed consent, (c) absence from the scheduled appointment for measurements, (d) presence of non-communicable diseases (NCDs), such as cardiovascular diseases, cancer, neurodegenerative diseases, and others, (e) food allergies, and (f) pregnancy or breastfeeding.

### 2.2. Sociodemographic Characteristics

Participants self-reported their sociodemographic characteristics, including age, gender, educational level, and marital status, during face-to-face interviews, which were conducted by medical and nursing staff, nutritionists, and dietitians in a private and relaxed setting to minimize recall bias. Educational level was categorized into three groups: (a) primary education, (b) secondary education, and (c) university studies.

### 2.3. Anthropometric Measurements

Anthropometric measurements were performed by one trained dietitian. Prior to the scheduled appointment, all participants were informed to abstain from any food or liquid intake for three hours prior to measurements, as well as any form of physical activity for 24 h. Height was measured to the nearest 0.5 cm using a stadiometer (HR-001, ΤAΝΙΤA, Manchester, UK). Body weight was measured to the nearest 0.1 kg with the use of a calibrated digital scale (SECA 876, SECA, Hamburg, Germany), with participants wearing light clothing and no shoes. Body mass index (BMI) was calculated as body weight divided by the square of height (kg/m^2^). Waist circumference was measured with a stretch-resistant tape over the naked skin or underwear, after a normal expiration, with arms relaxed at the sides under the midline of the participant’s armpit, at the midpoint between the lower part of the last rib and the top of the hip. Waist circumference was measured with an accuracy of 0.1 cm, two times, and an average value was recorded. Hip circumference was measured with the same tape over the naked skin or underwear, with the arms relaxed at the sides and at the maximum circumference over the hips. Hip circumference was also measured twice with an accuracy of 0.1 cm, and an average value was recorded.

For the measurement of body composition (i.e., body fat, muscle mass, and total body water), the Bioelectrical Impedance Analysis (BIA) method was used, with a calibrated Bioimpedance Analyser (BODYSTAT 1500, BODYSTAT, Douglas, Isle of Man, UK). Resting heart rate and blood pressure were assessed in the seated position using an electronic blood pressure monitor (Omron, Hoffman Estates, IL, USA), following a 10 min rest period. The International Obesity Task Force (IOTF) reference was utilized to define normal weight, overweight and obesity. Finally, physical activity in terms of engagement in leisure-time exercise was assessed (yes/no), as well as the number of hours of exercise per week.

### 2.4. Nutritional Assessment

Nutritional intake was reported through three 24 h diet recalls of food intake over three days, which included a Wednesday or Friday (during which the fasters obeyed fasting), another weekday, and a weekend day [27], a monthly food frequency questionnaire (FFQ), with 140 questions, was used to assess the participants’ dietary habits, and a validated questionnaire on eating habits and supplement use among other details [28]. The analysis depended on the mean of the 24 h diet recalls and was combined with the answers from the FFQs. Recommended Dietary Allowance (RDA) values were used to compare with each participant’s reported intake value. RDA is defined as the average daily level of intake sufficient to meet the nutrient requirements of nearly all (97–98%) healthy individuals; often used to plan nutritionally adequate diets for individuals.

Regarding the analysis of participants’ responses, food intake records were analyzed using the Food Processor v11.7 Nutrition Analysis software. Food intake was analyzed by grouping individual food items into broader food categories. The results are presented as portions per week for each food category.

### 2.5. Blood Pressure and Blood Analysis

Six milliliters of venous blood were drawn from a forearm vein while participants were in a seated position, between 8:00 and 10:00 a.m., following a 12 h overnight fast. The blood samples were collected in plain tubes without anticoagulant, allowed to clot at room temperature, and centrifuged at 1500× *g* for 10 min. Serum was then separated, aliquoted into Eppendorf tubes, and stored at −80 °C until analysis.

Serum calcium was measured using the Arsenazo III method on a Mindray BS-300 Chemistry Analyzer (Mindray Bio-Medical Electronics Co., Ltd, Shenzhen, China). Serum 25-hydroxyvitamin D levels were determined using a chemiluminescent microparticle immunoassay (CMIA) on an Abbott Architect i2000SR analyzer (Abbott Laboratories, Abbott Park, IL, USA). Urea, used as an index of protein intake, was measured via the urease–glutamate dehydrogenase method on the Mindray BS-300 Chemistry Analyzer. The coefficients of variation for calcium, vitamin D, and urea were 5%, 7%, and 3%, respectively. All analyses were performed in a laboratory participating in a nationwide external quality control program.

The blood pressure was measured with an electronic BP monitor (Omron, Hoffman Estates, IL, USA), and waist circumference was measured with an SECA body girth tape (SECA 201, Hamburg, Germany).

### 2.6. Statistical Analysis

Statistical analysis was performed using SPSS software version 29.0 (IBM Corp., Armonk, NY, USA). Descriptive statistics were used to summarize demographic and clinical characteristics. The normality of data distribution was assessed using the Kolmogorov–Smirnov test. Depending on the distribution, either the independent samples t-test or the Mann–Whitney U test was applied for group comparisons. Participants were classified into two BMI categories: overweight (BMI 25.0–29.9 kg/m^2^) and obesity (BMI ≥ 30.0 kg/m^2^). A *p* value of <0.05 was considered indicative of statistical significance.

## 3. Results

A total of 228 participants were included in the study. Their characteristics are described in Table 1. Fasters and non-fasters had a comparable profile in terms of demographic and BMI status, although non-fasters declared statistically significantly more hours of physical activity per week.

The analysis of nutrient intake values is presented in Table 2, with comparisons between fasters and non-fasters in subgroups with overweight and obesity. Non-fasters were found to consume higher amounts of calories, sugar, dietary protein, fats (saturated and polyunsaturated), and cholesterol.

Table 3 summarizes participants’ nutrient intake levels compared to the Recommended Dietary Allowances (RDA). Analysis of %RDA values showed that fasters had significantly lower intakes of vitamin A, vitamins B (particularly B2, B3, B6, B12, folate, pantothenic acid), iron, phosphorus, sodium, zinc, and calcium compared with non-fasters.

Comparisons of food group intake between fasters and non-fasters in subgroups with overweight and obesity are presented in detail in Table 4. Fasters reported significantly higher consumption of fish (especially in the obesity group), legumes (in the overweight group), fruits and juice, tahini (thick paste made from sesame seeds), ice pops/sorbet, ntolmadakia without meat, and taramas (Greek fish roe dip), as well as special salads. In contrast, non-fasters consumed significantly higher amounts of meat, dairy products, and alcohol, with differences more pronounced among participants with obesity.

The results of lab tests are presented in Table 5. Phosphorus levels were higher in all non-fasters regardless of their BMI status. Folic acid levels were higher in all fasters compared to non-fasters and among the individuals with overweight. Fasting glucose levels were higher in non-fasting participants both in the total study population and within the subgroup with overweight.

Table 6 presents the results from anthropometric measurements comparison between fasters and non-fasters. Diastolic blood pressure was statistically higher in non-fasters in all subgroup comparisons, while systolic blood pressure was also statistically significantly higher in the total group of non-fasters (and in the non-fasters with overweight).

## 4. Discussion

This study is among the limited published works investigating the effects of COC fasting practices on participants’ weight and nutritional status. Particular emphasis has been placed on analyzing BMI status to explore potential interaction effects between these two factors.

Concerning macronutrient reported intake, non-fasters participants in total were found to consume more saturated and polyunsaturated fat. The analysis showed significant differences in fat consumption among participants with overweight. Dietary fat is a crucial environmental factor that may interact with genetic predisposition to influence obesity risk [28,29]. Excessive fat intake has been linked to obesity [30,31,32], especially saturated fat [33]. The profile of macronutrient consumption indicates restriction of total protein among fasters. This finding is consistent with conclusions from similar studies [6,13,21]. Dietary fiber, though, was not consumed in higher proportions among fasters from our sample, which is a finding that does not agree with available data from other published studies, possibly associated with the inclusion of participants with increased BMI.

Considering micronutrient intake, non-fasters were found to consume higher amounts of micronutrients than fasters in our sample, even for vitamin B and folate, which seem to be overconsumed during fasting in other studies [3,4,14]. Iron and calcium consumption were found to be higher in non-fasters in total and among participants with obesity compared to fasters. Although previous studies have suggested that fasting may not affect iron status and does not appear to significantly increase the risk of iron deficiency in individuals with normal iron levels [9,33], our findings may suggest a potential risk for individuals who choose COC fasting, as they may be more susceptible to iron deficiency. As high levels of vitamin C enhance the absorption of iron, a synergistic effect of vitamin C on the adequacy of iron stores could be hypothesized, which is in accordance with previous findings [13,19].

Non-fasters were more likely to engage in exercise of higher duration compared to fasters. However, this level of engagement in physical activity was not sufficient to lower blood pressure among non-fasters, as diastolic blood pressure was significantly higher compared to fasters, while systolic blood pressure was elevated both in the total sample and specifically among overweight non-fasters participants.

The above findings highlight both the potential benefits and risks of COC fasting in relation to nutrient intake. While fasters demonstrated lower fat intake, which could be protective against obesity, their reduced consumption of key micronutrients such as vitamin B12, folate, calcium, and iron may raise concerns about long-term adequacy, particularly in vulnerable subgroups such as individuals with obesity. The apparent discrepancy with previous studies regarding dietary fiber suggests that adherence to fasting practices may vary across populations, and that food choices within fasting periods play a critical role in shaping nutrient profiles. Moreover, the observation that fasters exhibited lower blood pressure despite reporting lower levels of physical activity may indicate additional protective mechanisms of fasting—possibly mediated through improved dietary quality, reduced sodium, and saturated fat intake, or enhanced antioxidant status [34]. Taken together, these results underscore the complex interplay between diet composition, lifestyle factors, and health outcomes in the context of religious fasting and point to the need for tailored nutritional guidance to maximize benefits while preventing deficiencies.

The similar prevalence of overweight and obesity among fasters and non-fasters indicates that religious fasting, as practiced in this population, may not be sufficient to influence body weight outcomes, although fasters had lower fat intake. This may be attributed to the long-term focus on food quality rather than quantity during fasting periods, combined with a lack of portion control and overall dietary restraint, which can lead to excessive caloric intake despite the avoidance of certain food groups.

Christian Orthodox fasting has been linked to healthy choices [13,19]. Our findings support this opinion as they were found to consume higher amounts of healthy foods such as fish, fruit and vegetables. Furthermore, the lab test results revealed possible healthier tendencies with lower fasting glucose levels, lower transaminase levels, and lower creatinine and urea levels.

This study has certain limitations. Firstly, selection bias may be present, as all subjects were volunteers, and adherence to COC fasting could only be assessed through self-reported data. The 24 h dietary recall and the FFQ methods, although widely used to provide valuable insights into the nutritional habits of a population, may result in over- or underestimation of macro- and micronutrient intake and consequently lead to inaccuracies in dietary assessment [35]. Additionally, given the cross-sectional design of the study, firm conclusions based on causal associations cannot be established. Although it is advisable to report not only the absolute intake of carbohydrates, proteins, and lipids, but also their proportion of total energy intake, our dataset did not allow for the calculation of macronutrients as a proportion of total energy intake. Also, the intensity of leisure-time exercise was not assessed, which may limit the interpretation of the physical activity findings. Finally, the study sample, although one of the largest among similar studies, the inclusion of even more participants could lead to even more robust conclusions.

Among the strengths of this study is its robust design, which adheres to all ethical standards. Additionally, the inclusion of a sufficiently large non-fasting group, age-matched to the fasting group, allows for meaningful and comprehensive analyses.

## 5. Conclusions

In conclusion, this study suggests that COC fasting may influence dietary intake and certain biochemical parameters, with fasters reporting lower fat consumption and showing more favorable blood pressure and laboratory profiles. However, no clear effect on body weight outcomes was observed, as the prevalence of overweight and obesity was similar between fasters and non-fasters. Our findings indicate that COC fasting may confer certain cardiometabolic benefits, particularly through reduced fat intake and improved biochemical markers, but may also predispose to micronutrient inadequacies. Further large-scale cohort studies are needed to clarify the potential causal relationship and provide more tailored recommendations for practices associated with COC fasting in specific BMI populations.

## Figures and Tables

**Table 1:** Characteristics of the participants included in the study.

Characteristic	Fasters (Ν = 121) Mean ± SD/n (%)	Non-Fasters (Ν = 107) Mean ± SD/n (%)	*p*
Age (years)	49.7 ± 15.3	49.1 ± 15.9	0.918
Gender Male Female	42 (34.7) 79 (65.3)	52 (48.5) 55 (51.5)	0.062
Higher Educational Level achieved Primary school Secondary school University	9 (7.5) 39 (32.2) 73 (60.3)	15 (14) 32 (30) 60 (56)	0.467
Marital Status Married Not Married Divorced Widowers	86 (71) 32 (26.5) 3 (2.5) 0	73 (68.2) 30 (28) 2 (1.8) 2(1.8)	0.349
Leisure-time exercise Yes No	33 (27.3) 88 (72.7)	64 (59.8) 43 (40.2)	0.039 *
Leisure-time exercise (Hours weekly)	1.3 (3.7)	1.9 (2.8)	0.023 *
BMI status Overweight Obese (≥30)	76 (62.8) 45 (37.2)	69 (64.5) 38 (35.5)	0.793
BMI (kg/m^2^) Overweight Obese (≥30)	29.4 ± 3 27.5 ± 1.4 32.6 ± 2.5	29.7 ± 3.5 27.6 ± 2.2 33.1 ± 2.6	0.761

The *p* values demonstrating a significant difference are marked with *.

**Table 2:** Comparison of nutrient intake between fasters and non-fasters in subgroups with overweight (N = 145) and obesity (N = 83), as well as in the total study sample (N = 228).

Nutrient/Intake	Overweight (N = 145)			Obesity (N = 83)			All (N = 228)		
	Fasters (n = 76)	Non-Fasters (n = 69)	*p*	Fasters (n = 45)	Non-Fasters (n = 38)	*p*	Fasters (n = 121)	Non-Fasters (n = 107)	*p*
	Mean ± SD			Mean ± SD			Mean ± SD		
Protein (g)	51.04 ± 17.82	60.24 ± 22.52	0.007 *	51.45 ± 19.84	62.51 ± 20.42	0.015 *	51.19 ± 18.51	61.05 ± 21.73	<0.001 *
Carbohydrates (g)	153.04 ± 50.33	168.14 ± 66.10	0.137	152.00 ± 50.93	165.21 ± 58.07	0.358	152.6 ± 50.3	169 ± 63	0.137
Dietary fiber	20.9 ± 8.3	21.9 ± 10.9	0.748	21.8 ± 9.3	24.4 ± 13.6	0.516	21.2 ± 8.6	22.8 ± 11.9	0.748
Soluble fiber	2 ± 1.5	2.2 ± 1.5	0.843	1.7 ± 1.1	2 ± 1.5	0.342	2 ± 1.4	2 ± 1.5	0.843
Sugar-total	45.9 ± 28.5	57.3 ± 32.1	0.013 *	46 ± 22.4	48.5 ± 24	0.658	46 ± 26.3	54.2 ± 29.7	0.036 *
Monosacharides	18.1 ± 12.9	16.6 ± 15	0.183	14.8 ± 9.3	12.6 ± 11.4	0.071	16.8 ± 11.8	15.2 ± 13.9	0.043 *
Disaccharides (g)	13.25 ± 10.65	16.04 ± 10.72	0.056	11.12 ± 8.13	14.31 ± 11.16	0.107	12.45 ± 9.81	15.43 ± 10.86	0.013 *
Fat (g)	78.54 ± 25.88	89.12 ± 30.10	0.015 *	89.55 ± 28.36	95.08 ± 25.61	0.335	82.63 ± 27.25	91.24 ± 28.60	0.015 *
Saturated Fat (g)	20.00 ± 8.23	24.10 ± 9.80	0.005 *	23.10 ± 10.39	26.45 ± 11.22	0.233	21.15 ± 9.17	24.94 ± 10.34	0.005 *
Monosaturated fat (g)	41.5 ± 16.1	44.6 ± 16.6	0.181	46.2 ± 16.1	46.7 ± 14.1	0.549	43.3 ± 16.2	45.4 ± 15.8	0.181
Polyunsaturated Fat (g)	8.57 ± 3.11	10.17 ± 4.74	0.032 *	9.37 ± 3.57	10.65 ± 6.02	0.541	8.87 ± 3.30	10.34 ± 5.21	0.032 *
Trans fatty (g)	0.63 ± 0.54	0.87 ± 1.78	0.344	0.5 ± 0.34	0.76 ± 0.66	0.065	0.58 ± 0.48	0.83 ± 1.5	0.344
Cholesterol (mg)	132.29 ± 84.03	174.64 ± 82.80	<0.001 *	143.9 ± 103.5	180.3 ± 104.2	0.001 *	136.63 ± 91.51	176.66 ± 90.56	<0.001 *
Omega-3 Fatty Acid (g)	0.63 ± 0.37	0.72 ± 0.45	0.119	0.73 ± 0.66	0.74 ± 0.47	0.462	0.66 ± 0.49	0.73 ± 0.45	0.119
Omega-6 Fatty Acids (g)	4.86 ± 2.89	5.91 ± 4.19	0.108	4.89 ± 2.10	5.94 ± 3.94	0.615	4.87 ± 2.62	5.92 ± 4.09	0.108
Alcohol (g)	2.06 ± 5.57	4.54 ± 9.37	0.006 *	2.02 ± 5.41	4.00 ± 8.69	0.029 *	2.06 ± 5.6	4.3 ± 9.3	0.006 *
Vitamin A (RE)	415.77 ± 346.62	603.33 ± 792.96	0.401	300.02 ± 223.01	549.42 ± 496.24	0.008 *	372.72 ± 310.62	584.19 ± 700.00	0.019 *
Thiamine (B1)	1.24 ± 1.16	1.24 ± 0.51	0.231	1.20 ± 0.95	1.16 ± 0.89	0.215	1.18 ± 0.96	1.22 ± 0.51	0.141
Riboflavin B2 (mg)	1.11 ± 0.45	1.43 ± 0.70	0.006 *	1.02 ± 0.44	1.38 ± 0.70	0.017 *	1.07 ± 0.45	1.41 ± 0.69	<0.001 *
Niacin B3 (mg)	9.55 ± 5.59	11.30 ± 6.05	0.069	8.05 ± 4.23	10.37 ± 6.38	0.071	8.99 ± 5.16	10.97 ± 6.16	0.010 *
Niacin equiv. (mg)	15.66 ± 8.05	19.23 ± 9.61	0.03 *	13.70 ± 7.16	18.99 ± 9.95	0.011 *	14.9 ± 7.8	19.1 ± 9.7	<0.001 *
Vitamin B6 (mg)	1.17 ± 0.32	1.45 ± 0.58	<0.001 *	1.17 ± 0.12	1.42 ± 0.36	0.086	1.17 ± 0.42	1.43 ± 0.58	<0.001 *
Vitamin B12 (mcg)	2.41 ± 2.40	4.56 ± 5.41	<0.001 *	2.47 ± 3.38	2.89 ± 2.38	0.078	2.43 ± 2.80	3.97 ± 4.63	0.014 *
Vitamin E (mg)	6.08 ± 3.23	7.39 ± 7.42	0.336	6.02 ± 2.36	8.50 ± 6.46	0.522	6.06 ± 3.23	7.78 ± 7.09	0.336
Vitamin C (mg)	228 ± 352.2	141.7 ± 222.5	0.148	143.9 ± 170	95.9 ± 64.9	0.300	196.8 ± 299	125.4 ± 183	0.148
Vitamin D (mcg)	1.7 ± 1.5	2.7 ± 3	0.133	1.96 ± 1.7	1.98 ± 3.80	0.088	1.80 ± 2.6	2.46 ± 2.68	0.029 *
Folate (mcg)	182.83 ± 86.25	218.98 ± 117.41	0.061	159.42 ± 64.25	221.58 ± 122.12	0.010 *	174.12 ± 79.32	219.90 ± 118.53	0.002 *
Calcium (mg)	569.4 ± 259	703.8 ± 380	0.046 *	567.5 ± 280	770.7 ± 398	0.018 *	568 ± 266	727.6 ± 386	0.002 *
Copper (mg)	0.65 ± 0.26	0.73 ± 0.40	0.349	0.60 ± 0.26	0.76 ± 0.33	0.026 *	0.63 ± 0.26	0.74 ± 0.38	0.041 *
Iron (mg)	9.59 ± 3.37	10 ± 4.1	0.748	10.00 ± 4.09	11.49 ± 6.18	0.043 *	9.36 ± 3.18	10.58 ± 4.95	0.148
Phosphorus (mg)	755.73 ± 259.84	934.81 ± 392.63	0.064	725.64 ± 284.48	989.17 ± 416.13	0.004 *	755.73 ± 259.84	934.81 ± 392.63	<0.001 *
Zinc (mg)	6.18 ± 2.63	7.84 ± 3.47	<0.001 *	6.30 ± 3.05	8.61 ± 3.81	0.006 *	6.23 ± 2.78	8.12 ± 3.60	<0.001 *
Magnesium (mg)	177.29 ± 65.23	185.49 ± 72.53	0.125	153.38 ± 46.95	183.72 ± 67.85	0.044 *	168.40 ± 53.38	184.86 ± 70.58	0.125
Pantothenic acid (mg)	2.13 ± 0.89	2.65 ± 1.35	0.036 *	1.86 ± 0.89	2.73 ± 1.17	0.001 *	2.03 ± 0.90	2.68 ± 1.29	<0.001 *
Manganese (mg)	1.36 ± 0.89	1.45 ± 1.04	0.207	0.97 ± 0.50	1.41 ± 0.91	0.023 *	1.27 ± 0.9	1.38 ± 0.8	0.149

The *p* values demonstrating a significant difference are marked with *.

**Table 3:** Comparisons of %RDA intake for different micronutrients between fasters and non-fasters in subgroups with overweight (N = 145) and obesity (N = 83), as well as in the total study sample (N = 228).

RDA% Nutrient Intake	Overweight (N = 145)			Obesity (N = 83)			All (N = 228)		
	Fasters (n = 76)	Non-Fasters (n = 69)	*p*	Fasters (n = 45)	Non-Fasters (n = 38)	*p*	Fasters (n = 121)	Non-Fasters (n = 107)	*p*
	Mean ± SD			Mean ± SD			Mean ± SD		
Vitamin A	54.3 ± 47	76.8 ± 108	0.404	38.9 ± 30.5	68.3 ± 58.9	0.005 *	48.6 ± 42.2	73.8 ± 93.6	0.029 *
Vitamin B1	98.1 ± 31.4	107.3 ± 42.8	0.324	94.4 ± 34.3	103.3 ± 41.9	0.323	96.7 ± 32.4	105.9 ± 42.3	0.176
Viatmin B2	95 ± 38.7	117.3 ± 54.3	0.011 *	86.5 ± 37.9	115 ± 41.9	0.017 *	91.8 ± 38.5	116.5 ± 53.9	<0.001 *
Vitamin B3	64.8 ± 37.4	74.2 ± 37.8	0.100	54.4 ± 28.4	69.3 ± 42.5	0.071	60.9 ± 34.6	72.5 ± 39.4	0.018 *
Niacin equiv.	107.1 ± 54.5	128.9 ± 61.6	0.006 *	93.04 ± 48.9	129.4 ± 65.4	0.006 *	101.9 ± 52.7	129 ± 62.7	0.001 *
Vitamin B6	85.2 ± 30.9	103.4 ± 44.4	0.005 *	82.7 ± 28.2	97.7 ± 38	0.127	84.3 ± 29.8	101.4 ± 42.1	0.002 *
Vitamin B12	101.04 ± 100.5	190.6 ± 225.3	0.002 *	103.4 ± 141	121.4 ± 99	0.082	101.9 ± 116.6	166 ± 192.8	<0.001 *
Vitamin C	283 ± 413	169 ± 249	0.133	176.5 ± 196.9	119.4 ± 75.7	0.375	243.7 ± 352	151.5 ± 206	0.084
Vitamin D	24.8 ± 22.6	38.9 ± 44.7	0.147	24.1 ± 38.9	26.4 ± 26.2	0.177	24.6 ± 29.6	34.5 ± 39.5	0.057
Vitamin E equiv.	41.8 ± 25.7	49.9 ± 49.4	0.543	40.2 ± 15.7	59.9 ± 45	0.289	41.2 ± 22.4	53.5 ± 47.9	0.234
Folate	45.7 ± 21.6	54.9 ± 29.4	0.059	39.9 ± 16	55.4 ± 30.5	0.012 *	43.6 ± 19.8	55 ± 29.7	0.003 *
Pantothenic	42.4 ± 17.2	52.6 ± 26.8	0.034 *	37.2 ± 17.8	54.5 ± 23.4	0.001 *	40.5 ± 17.5	53.3 ± 25.5	<0.001 *
Ca	52.03 ± 23.3	64.4 ± 35.2	0.043 *	50.9 ± 26	68.6 ± 37.4	0.025 *	51.6 ± 24.2	65.9 ± 35.8	0.003 *
Cu	66 ± 26.7	74.8 ± 40.7	0.336	61.3 ± 26	77.2 ± 33	0.022 *	64.3 ± 26.5	75.6 ± 38	0.033 *
Fe	94.8 ± 51.9	109.9 ± 55.2	0.076	102.3 ± 42.9	131.8 ± 84.6	0.153	97.6 ± 48.7	117.7 ± 67.6	0.030 *
Mg	49.8 ± 18.2	51.6 ± 17	0.373	43 ± 12.7	50.6 ± 17.1	0.049 *	48.4 ± 16	50 ± 17.7	0.659
Mn	73.1 ± 53.8	64.5 ± 43.2	0.596	48.8 ± 25.2	64.5 ± 36.5	0.052	64 ± 46.7	64.5 ± 40.7	0.489
P	109.6 ± 36.4	129.3 ± 54	0.056	103.6 ± 40.5	141.3 ± 59.5	0.003 *	107.4 ± 37.9	133.5 ± 56.1	<0.001 *
K	62.9 ± 20.3	66.3 ± 24.7	0.710	58 ± 17.2	61.6 ± 20.9	0.434	61 ± 19.3	64.6 ± 23.4	0.424
Se	86.3 ± 48.5	93.7 ± 51.8	0.354	77.2 ± 48.7	96.4 ± 53.8	0.106	82.9 ± 48.6	94.7 ± 52.3	0.087
Na	32.5 ± 26.3	39.3 ± 21.3	0.019 *	38.8 ± 40.7	40.7 ± 33.7	0.664	34.8 ± 32.5	39.8 ± 26.3	0.03 *
Zn	68.5 ± 29.8	80.7 ± 33	0.018 *	69.6 ± 35.4	91.9 ± 41.8	0.015 *	68.9 ± 31.8	84.7 ± 36.6	<0.001 *

The *p* values demonstrating a significant difference are marked with *.

**Table 4:** Comparisons of food groups intake between fasters and non-fasters in subgroups with overweight (N = 145) and obesity (N = 83), as well as in the total study sample (N = 228).

	Overweight (N = 145)			Obesity (N = 83)			All (N = 228)		
Food Category	Fasters (n = 76)	Non-Fasters (n = 69)	*p*	Fasters (n = 45)	Non-Fasters (n = 38)	*p*	Fasters (n = 121)	Non-Fasters (n = 107)	*p*
	Mean ± SD	Mean ± SD		Mean ± SD	Mean ± SD		Mean ± SD	Mean ± SD	
Meat (140 g)	1.59 ± 0.26	1.68 ± 0.24	0.064	1.56 ± 0.26	1.70 ± 0.28	0.031 *	1.58 ± 0.26	1.69 ± 0.25	0.005 *
Snails (10 g)	1.07 ± 0.36	1.04 ± 0.27	0.619	1.09 ± 0.42	1.05 ± 0.33	0.661	1.07 ± 0.36	1.04 ± 0.27	0.500
Fish (140 g)	1.78 ± 0.45	1.71 ± 0.47	0.332	1.72 ± 0.35	1.58 ± 0.46	0.033 *	1.75 ± 0.41	1.66 ± 0.47	0.052
Legumes (150 g)	1.78 ± 0.47	1.61 ± 0.30	0.041 *	1.59 ± 0.29	1.60 ± 0.28	0.984	1.70 ± 0.42	1.60 ± 0.29	0.094
Dairy	1.54 ± 0.26	1.55 ± 0.20	0.604	1.43 ± 0.23	1.55 ± 0.25	0.012 *	1.5 ± 0.25	1.55 ± 0.22	0.039 *
Cream	1.36 ± 0.47	1.2 ± 0.36	0.051	1.27 ± 0.37	1.23 ± 0.28	0.941	1.32 ± 0.44	1.21 ± 0.33	0.107
Bread and bread products (40 g)	1.73 ± 0.36	1.76 ± 0.36	0.904	1.68 ± 0.24	1.79 ± 0.35	0.154	1.71 ± 0.31	1.76 ± 0.36	0.403
Pies	2.41 ± 0.96	2.59 ± 1.09	0.105	2.44 ± 0.89	2.57 ± 1.21	0.585	2.41 ± 0.96	2.59 ± 1.08	0.351
Sugar, Honey & Marmalades	2.70 ± 0.98	3.02 ± 089	0.052	2.88 ± 1.13	2.67 ± 0.96	0.410	2.77 ± 1.04	2.90 ± 0.93	0.311
Pastitsio/mousaka	1.91 ± 0.97	2.10 ± 0.97	0.387	1.84 ± 0.95	2.11 ± 0.99	0.184	1.91 ± 0.97	2.10 ± 0.97	0.131
Taramas	1.64 ± 0.94	1.13 ± 0.48	<0.001 *	1.6 ± 1.25	1.24 ± 0.59	0.248	1.63 ± 1.06	1.17 ± 0.53	<0.001 *
Ntolmadakia (with meat & rice)	1.78 ± 0.97	1.80 ± 0.99	0.687	1.76 ± 1.01	1.78 ± 1.03	0.809	1.76 ± 0.97	1.80 ± 0.99	0.635
Ntolmadakia (without meat)	2.13 ± 0.97	1.87 ± 0.90	0.210	2.29 ± 1.08	1.92 ± 0.86	0.103	2.12 ± 0.97	1.87 ± 0.85	0.047 *
Pizza	2.09 ± 0.99	2.08 ± 0.97	0.423	1.89 ± 0.98	2.11 ± 0.94	0.240	2.09 ± 0.99	2.08 ± 0.97	0.949
Potatoes (150 g)	2.2 ± 0.59	2.20 ± 0.52	0.875	2.25 ± 0.62	2.19 ± 0.48	0.635	2.21 ± 0.6	2.20 ± 0.50	0.872
Rice (220 g)	1.72 ± 0.44	1.74 ± 0.39	0.545	1.65 ± 0.41	1.63 ± 0.38	0.872	1.69 ± 0.43	1.70 ± 0.40	0.652
Vegetables (180 g)	2.90 ± 0.63	2.84 ± 0.58	0.404	2.68 ± 0.65	2.72 ± 0.63	0.658	2.82 ± 0.65	2.80 ± 0.60	0.714
Fruits & Juice (200 g)	2.48 ± 0.66	2.30 ± 0.59	0.108	2.27 ± 0.59	2.02 ± 0.51	0.071	2.4 ± 0.65	2.20 ± 0.58	0.024 *
Soft & Energy Drinks (250 mL)	1.14 ± 0.25	1.19 ± 0.35	0.788	1.18 ± 0.32	1.20 ± 0.26	0.248	1.15 ± 0.28	1.20 ± 0.33	0.337
Oils and Butter	2.14 ± 0.51	2.14 ± 0.54	0.973	2.13 ± 0.57	2.17 ± 0.43	0.532	2.13 ± 0.53	2.16 ± 0.50	0.730
Sweet Snacks (80 g)	1.69 ± 0.49	1.56 ± 0.42	0.138	1.71 ± 0.42	1.58 ± 0.51	0.116	1.7 ± 0.46	1.57 ± 0.45	0.029 *
Salty Snacks (40 g)	1.43 ± 0.79	1.33 ± 0.66	0.740	1.49 ± 0.82	1.35 ± 0.75	0.441	1.45 ± 0.80	1.34 ± 0.69	0.449
Coffee and Beverages (250 mL)	1.82 ± 0.52	1.91 ± 0.42	0.147	1.85 ± 0.45	1.97 ± 0.43	0.694	1.84 ± 0.50	1.94 ± 0.42	0.185
Alcohol (40 g)	1.39 ± 0.44	1.76 ± 0.53	<0.001 *	1.37 ± 0.43	1.66 ± 0.65	0.046 *	1.39 ± 0.43	1.72 ± 0.57	<0.001 *
Cereals	1.32 ± 0.44	1.48 ± 0.68	0.407	1.28 ± 0.43	1.20 ± 0.40	0.408	1.3 ± 0.44	1.39 ± 0.61	0.860
Tahini (Sesame paste) (17 g)	1.99 ± 1.22	1.67 ± 1.43	0.005 *	1.62 ± 0.91	1.19 ± 0.52	0.010 *	1.85 ± 1.12	1.50 ± 1.21	<0.001 *
Ice pop/Sorbe	1.37 ± 0.79	1.20 ± 0.63	0.119	1.42 ± 082	1.11 ± 0.46	0.068	1.39 ± 0.80	1.17 ± 0.58	0.019 *
Special salads (smashed eggplant, tzatziki, etc.)	1.80 ± 0.93	2.17 ± 1.37	0.039 *	1.58 ± 0.89	2.16 ± 1.14	0.018 *	1.72 ± 0.92	2.17 ± 1.13	0.002 *
Tomato Sause	1.65 ± 0.95	1.75 ± 1.13	0.745	1.56 ± 0.84	1.43 ± 0.77	0.595	1.62 ± 0.90	1.64 ± 1.05	0.981
Mustard	2.64 ± 1.76	2.32 ± 1.57	0.259	2.36 ± 1.62	2.22 ± 1.5	0.935	2.53 ± 1.71	2.28 ± 1.53	0.348
Water (250 mL)	5.96 ± 0.20	5.88 ± 0.63	0.594	5.96 ± 0.20	5.95 ± 0.22	0.863	5.96 ± 0.20	5.91 ± 0.53	0.595

The *p* values demonstrating a significant difference are marked with *.

**Table 5:** Comparisons of blood test results between fasters and non-fasters in overweight (N = 145) and obese (N = 83) subgroups, as well as in the total study sample (N = 228).

	Overweight (N = 145)			Obese (N = 83)			All (N = 228)		
Lab Test	Fasters (n = 76)	Non-Fasters (n = 69)	*p*	Fasters (n = 45)	Non-Fasters (n = 38)	*p*	Fasters (n = 121)	Non-Fasters (n = 107)	*p*
	Mean ± SD	Mean ± SD		Mean ± SD	Mean ± SD		Mean ± SD	Mean ± SD	
Fe (μg/dL)	91.1 ± 31.6	106.3 ± 44.9	0.051	104.1 ± 44.5	85.8 ± 36.5	0.098	95.9 ± 37.3	99 ± 43.1	0.614
Creatinine (mg/dL)	0.90 ± 0.18	0.98 ± 0.18	0.007 *	0.98 ± 0.17	0.92 ± 0.17	0.082	0.93 ± 0.18	0.96 ± 0.18	0.258
Urea (mg/dL)	28.4 ± 10.4	32.7 ± 10.7	0.007 *	33.2 ± 14	31.3 ± 10.2	0.982	30.2 ± 12	32.2 ± 10.5	0.041 *
Uric acid (md/dL)	4.6 ± 1.3	4.8 ± 1.4	0.138	5 ± 1.4	4.9 ± 1.4	0.467	4.84 ± 1.39	4.87 ± 1.37	0.431
Albumin/Globulin Ratio	6.85 ± 0.56	6.99 ± 0.56	0.195	6.92 ± 0.56	6.90 ± 0.58	0.482	6.87 ± 0.57	6.85 ± 0.57	0.167
Albumin (g/dL)	4.30 ± 0.21	4.39 ± 0.27	0.097	4.26 ± 0.29	4.30 ± 0.27	0.602	4.29 ± 0.24	4.35 ± 0.27	0.108
γ-GT (U/l)	19.9 ± 13.4	23.2 ± 22.7	0.528	26.3 ± 17.6	37.5 ± 80.2	0.923	22.3 ± 15.4	28.3 ± 51.2	0.616
Ca (mg/dL)	9.75 ± 0.54	9.8 ± 0.58	0.882	9.85 ± 0.84	9.86 ± 0.68	0.989	9.8 ± 0.67	9.8 ± 0.62	0.934
ALP (U/L)	67.5 ± 19.5	73.5 ± 27.8	0.480	72.7 ± 18.9	72.5 ± 32.8	0.421	69.7 ± 19.3	73.2 ± 29.6	0.979
Cholesterol (mg/dL)	194.3 ± 52.3	205.8 ± 51.6	0.143	202.1 ± 43.7	216 ± 48.1	0.356	197.6 ± 49.2	209.5 ± 50.4	0.089
Tg (mg/dL)	130.2 ± 75.1	148.2 ± 84.2	0.165	182.5 ± 125.5	200.9 ± 11.3	0.196	149.6 ± 99.8	166.9 ± 97.6	0.063
HDL (mg/dL)	54 ± 14.2	51.3 ± 16.9	0.404	45.1 ± 15.3	49.6 ± 11.4	0.077	50.8 ± 15.2	50.6 ± 15.2	0.888
LDL (mg/dL)	118.6 ± 54.8	115 ± 50.5	0.808	117.8 ± 64.9	113.8 ± 47.2	0.971	118.3 ± 58.5	114.6 ± 49.1	0.772
SGOT (U/L)	13.6 ± 8	15.2 ± 8.5	0.261	13.2 ± 8.2	15.7 ± 6.9	0.124	13.4 ± 8	15.5 ± 7.9	0.050 *
SGPT (U/L)	8.8 ± 6.6	10.9 ± 8.7	0.301	9.74 ± 11	12.8 ± 11.3	0.041	9.13 ± 8.5	11.6 ± 9.7	0.022 *
Vitamin D 25-OH (ng/mL)	18.5 ± 6.9	18.9 ± 5.6	0.434	16.6 ± 5.9	17.7 ± 6.5	0.596	17.8 ± 6.6	18.4 ± 5.9	0.270
Folic Aid (ng/mL)	4.4 ± 5.9	2.5 ± 3.1	0.004 *	2.94 ± 3	2.35 ± 3	0.105	3.9 ± 5.1	2.5 ± 3	0.001 *
Vitamin Β12 (pg/mL)	333.4 ± 363.8	331.6 ± 118.9	0.025 *	301.3 ± 124.7	304.3 ± 92.5	0.525	321.5 ± 297.8	321.9 ± 110.6	0.023 *
Insulin (μIU/mL)	4.5 ± 5.5	3.2 ± 2.9	0.283	6.23 ± 6.9	3.4 ± 3	0.007 *	5.14 ± 6.11	3.26 ± 2.9	0.015 *
Magnesium (mg/dL)	1.88 ± 0.19	1.75 ± 0.2	<0.001 *	1.80 ± 0.22	1.8 ± 0.20	0.502	1.85 ± 0.2	1.76 ± 0.2	0.001 *
C-Reactive Protein (mg/dL)	0.21 ± 0.30	0.20 ± 0.23	0.863	0.32 ± 0.25	0.38 ± 0.38	0.780	0.26 ± 0.29	0.26 ± 0.31	0.978
Phosphorus (mg/dL)	5 ± 1.7	5.8 ± 1.1	0.002 *	5 ± 1.8	6.3 ± 1.4	<0.001 *	5.04 ± 1.7	5.96 ± 1.24	0.000 *
Glucose (mg/dL)	81.8 ± 13.7	87.9 ± 16.1	0.017 *	90.9 ± 17.3	98.08 ± 30	0.052	85.2 ± 15.7	91.5 ± 22.5	0.003 *

The *p* values demonstrating a significant difference are marked with *.

**Table 6:** Comparisons of anthropometric measurements between fasters and non-fasters in overweight (N = 145) and obese (N = 83) subgroups, as well as in the total study sample (N = 228).

	Overweight (N = 145)			Obese (N = 83)			All (N = 228)		
	Fasters (n = 76)	Non-Fasters (n = 69)	*p*	Fasters (n = 45)	Non-Fasters (n = 38)	*p*	Fasters (n = 121)	Non-Fasters (n = 107)	*p*
Body Fat %	33 ± 7	32 ± 6.7	0.310	40.1 ± 6.3	39.8 ± 6.6	0.880	35.7 ± 7.6	34.8 ± 7.6	0.350
Fat Mass (kg)	24.8 ± 5.6	25 ± 4.8	0.750	37.3 ± 7.1	35.8 ± 6.5	0.360	29.4 ± 8.6	28.9 ± 7.5	0.750
Waist Circumference (cm)	92.7 ± 8.1	93.7 ± 8.3	0.470	106.1 ± 10	104.5 ± 8.3	0.440	97.7 ± 11	97.6 ± 9.7	0.920
Fat Free Mass (kg)	50.6 ± 9.3	54.4 ± 11.5	0.130	56 ± 11.2	54.9 ± 12.3	0.310	52.6 ± 10.4	54.6 ± 11.7	0.430
Hip Circumstance (cm)	101.1 ± 7.4	100.4 ± 5.9	0.500	107.6 ± 7.3	104.2 ± 8.5	0.030 *	103.5 ± 7.9	101.8 ± 7.1	0.040 *
Hip/Waist Circumstance	0.92 ± 0.09	0.94 ± 0.09	0.340	0.99 ± 0.11	1 ± 0.09	0.230	0.95 ± 0.10	0.96 ± 0.10	0.290
Abdominal Circumstance (cm)	85.1 ± 7.9	87.6 ± 8.8	0.080	100.4 ± 11	98 ± 8.16	0.140	90.8 ± 11.9	91.2 ± 9.9	0.380
Systolic BP (mmHg)	127.7 ± 11	132.4 ± 14	0.030 *	135.6 ± 12	138 ± 10.4	0.130	130.6 ± 12	134.4 ± 13.1	0.030 *
Diastolic BP (mmHg)	78.3 ± 8.5	82.1 ± 9.4	0.003 *	81.8 ± 7.5	85.8 ± 7.2	0.016 *	79.6 ± 8.3	83.4 ± 8.8	<0.001 *
Heart Rate (beats/min)	68.8 ± 10	69.6 ± 10.6	0.620	73.4 ± 8.2	72 ± 11	0.380	70.5 ± 9.7	70.5 ± 10.7	0.980

The *p* values demonstrating a significant difference are marked with *.

## Data Availability

The data presented in this study are available on request from the corresponding author due to ethical reasons.

## Abbreviations

The following abbreviations are used in this manuscript:

aPTT	Activated Partial Thromboplastin Time
BIA	Bioelectrical Impedance Analysis
FFQ	Food Frequency Questionnaire
BMI	Body Mass Index
COC	Christian Orthodox Church
INR	International Standardized Ratio
IOTF	International Obesity Task Force
MCV	Mean Corpuscular Volume
NCDs	Non-Communicable Diseases
RDA	Recommended Dietary Allowances
